# Synergy for Salmon: Study Spawns Insight into Pesticide Mixtures

**Published:** 2009-03

**Authors:** Bob Weinhold

Years of habitat degradation, overfishing, hatchery practices, and dam building have left U.S. wild salmon populations struggling to recuperate. Another potential threat that hasn’t been considered is the combined impact of multiple pesticides that are found in waterways. In a study of various pesticide mixtures, researchers found the presence of adverse effects that were synergistic, not just the additive effects anticipated under current regulations **[*EHP* 117:348–353; Laetz et al.]**. In light of the current findings, mixtures that have been considered relatively safe may pose more of a hazard to wildlife than was previously thought.

The researchers evaluated the effects of diazinon, malathion, chlorpyrifos, carbaryl, and carbofuran—which are among the most extensively used pesticides in California and the Pacific Northwest—in the brains of juvenile coho salmon. These chemicals inhibit the enzyme acetylcholinesterase (AChE), resulting in an accumulation of acetylcholine, which in turn can affect behavior and, ultimately, survival.

For each of 10 pairings of the 5 pesticides, concentrations were designed to elicit AChE reductions of 10%, 29%, or 50% (assuming the chemicals acted additively) for a total of 30 possible exposures. Other fish were exposed to single pesticides; none were tested for combinations of 3 or more chemicals.

Nearly every pairing inhibited AChE activity after the salmon were exposed over a 96-hour period. Synergistic inhibition was observed in 20 of the 30 combinations, producing anywhere from about 20% stronger inhibition than predicted by additive activity alone to more than 90% inhibition in 5 combinations. For 3 combinations, the salmon died within 24 hours. In contrast, there were no deaths among fish exposed to individual pesticides only.

The synergistic effects were almost uniformly more pronounced as the exposure increased. Nonetheless, even at lower, more environmentally relevant concentrations, the synergistic effects were significant for 4 of the 10 pairings. For some chemical combinations, the data suggest synergistic effects are possible at concentrations below the lowest levels used in the study.

In a 2007 study, more than 90% of water samples from urban, agricultural, and mixed-use streams contained 2 or more pesticides, residue from more than 1.2 billion pounds of pesticides applied each year. The investigators conclude that more studies of pesticide combinations must be done on live fish—especially since their results weren’t predicted by *in vitro* studies—and that more work is needed to determine the lower bounds for pesticide interactions. Furthermore, if synergistic effects occur at concentrations found in habitats supporting salmon stocks, which often include species designated as threatened or endangered, regulators may need to consider multichemical effects when setting exposure standards.

## Figures and Tables

**Figure:**
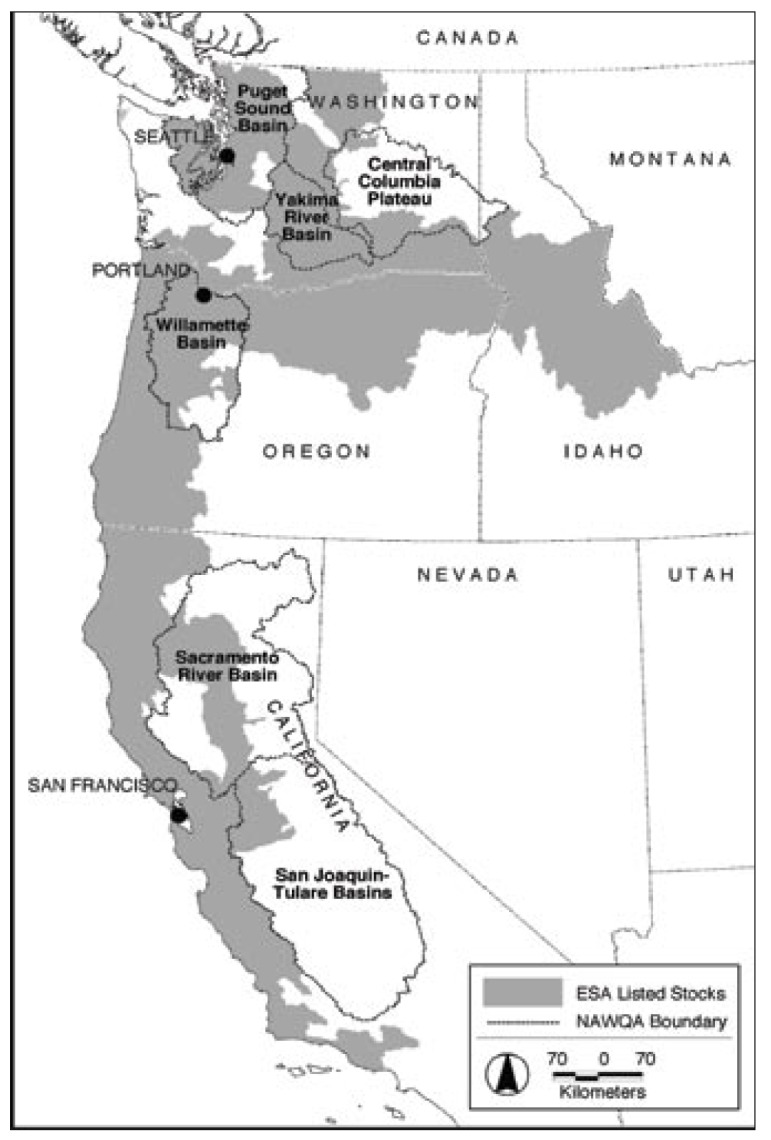
This map shows the overlap between the range of endangered salmon species (gray areas) and study areas where multiple pesticides have been measured in surface waters (dashed lines).

